# Shining a Light on Awareness: A Review of Functional Near-Infrared Spectroscopy for Prolonged Disorders of Consciousness

**DOI:** 10.3389/fneur.2018.00350

**Published:** 2018-05-22

**Authors:** Mohammed Rupawala, Hamid Dehghani, Samuel J. E. Lucas, Peter Tino, Damian Cruse

**Affiliations:** ^1^Centre for Doctoral Training in Physical Sciences for Health, University of Birmingham, Birmingham, United Kingdom; ^2^School of Computer Science, University of Birmingham, Birmingham, United Kingdom; ^3^School of Sport, Exercise and Rehabilitation Sciences, University of Birmingham, Birmingham, United Kingdom; ^4^School of Psychology, University of Birmingham, Birmingham, United Kingdom

**Keywords:** disorders of consciousness, functional near-infrared spectroscopy, electroencephalography, motor imagery, data fusion, brain–computer interface

## Abstract

Qualitative clinical assessments of the recovery of awareness after severe brain injury require an assessor to differentiate purposeful behavior from spontaneous behavior. As many such behaviors are minimal and inconsistent, behavioral assessments are susceptible to diagnostic errors. Advanced neuroimaging tools can bypass behavioral responsiveness and reveal evidence of covert awareness and cognition within the brains of some patients, thus providing a means for more accurate diagnoses, more accurate prognoses, and, in some instances, facilitated communication. The majority of reports to date have employed the neuroimaging methods of functional magnetic resonance imaging, positron emission tomography, and electroencephalography (EEG). However, each neuroimaging method has its own advantages and disadvantages (e.g., signal resolution, accessibility, etc.). Here, we describe a burgeoning technique of non-invasive optical neuroimaging—functional near-infrared spectroscopy (fNIRS)—and review its potential to address the clinical challenges of prolonged disorders of consciousness. We also outline the potential for simultaneous EEG to complement the fNIRS signal and suggest the future directions of research that are required in order to realize its clinical potential.

## Introduction

In the UK, every 3 minutes an individual is hospitalized due to a traumatic (e.g., fall, assault, motor vehicle accident) or non-traumatic (e.g., stroke, brain hemorrhage, anoxia) brain injury, equating to approximately 300,000 admissions per year.^1^ While many patients experience little or no long-term effects, a significant number of patients will develop a prolonged disorder of consciousness (PDOC), such as a vegetative state or minimally conscious state. Patients in a vegetative state [also known as unresponsive wakefulness syndrome (1)] are clinically awake, with eyes open and preserved reflexes, yet appear to be unaware of their surroundings or of themselves [for a detailed review of the PDOC states, please refer to Ref. (2)]. Patients in a minimally conscious state exhibit inconsistent but purposeful evidence of awareness, such as visual pursuit and following verbal commands (3).

Partial or full recovery following severe brain injury can in many cases involve transitioning between each of these states (4). The progression is generally smooth (4) and therefore the difficulty lies in accurately determining and diagnosing a patient in a single state using qualitative clinical assessment methods. The need to accurately detect awareness remains a thorough subject of research as misdiagnoses can lead to inappropriate healthcare decisions (5). Standardized behavioral assessments are the current “gold standard” for detecting signs of awareness (6, 7). However, as clinicians must rely on observable behaviors to determine a patient’s level of awareness, it is possible that a significant proportion of patients can be misdiagnosed if they are unable to produce purposeful behaviors due to a motor impairment. Indeed it has been estimated that 15% of patients (8) who meet the behavioral gold standard for vegetative state have a cognitive-motor dissociation (9) or covert awareness (10) that can only be detected with brain imaging.

In the first demonstration of covert command-following, Owen et al. asked a patient who fulfilled all clinical criteria for a diagnosis of vegetative state to undertake two motor imagery tasks in the functional magnetic resonance imaging (fMRI) scanner; the first involved playing a game of tennis and the second, a spatial navigation task, involved imagining visiting the rooms of her house (11). As is seen in healthy individuals when completing the same tasks, significant activity was observed in the patient’s supplementary motor area (SMA) while imagining playing tennis, and in the parahippocampal gyrus, the posterior parietal cortex, and the lateral premotor cortex (PMC) when imagining moving around her house. This brain imaging evidence of the patient following the commands indicated that she was aware, despite the fact that she was unable to demonstrate it with her behavior. Subsequently, by assigning each imagery task to a “yes” or “no” communication output, several patients have been able to answer a series of questions about themselves and their lives (12–15), hinting at the potential for brain–computer interfaces (BCIs) and assistive devices for this patient group. Here, a BCI is defined using the definition proposed by Wolpaw et al.: a device that “provides the brain with a new, non-muscular communication and control channel” [(16), p. 768]. In this context, a BCI serves to directly measure neural activity associated with the users’ intent and translate the recorded signals into corresponding control signals for BCI applications.

Despite the success of fMRI in the field of PDOC, the technology is limited because many patients’ reduced mobility requires them to be transported to advanced facilities that feature such equipment. Furthermore, fMRI systems are unsuitable for those with metallic implants, are highly sensitive to motion artifacts, and require patients to lay supine. A portable, inexpensive, and non-magnetic method for measuring the same hemodynamic response as measured by fMRI could be used to translate the successes of fMRI to the bedside.

The hemodynamic response is a collective term for the set of physiological responses that take place during the onset of neuronal activations. For example, the blood oxygenation level-dependent (BOLD) signal detected in fMRI systems are sensitive to changes in cerebral blood flow, cerebral metabolic rate of oxygen and cerebral blood volume (17). Increases in these elements during neural activation result in slight increases in the local magnetic resonance signal and thus small changes in the BOLD signal that can be detected by fMRI.

Functional near-infrared spectroscopy (fNIRS) is an alternative method to fMRI that similarly measures BOLD-like hemodynamic responses (18, 19). Furthermore, this method is portable, inexpensive, fast, non-invasive and has limited contraindications (18, 20, 21). Nevertheless, without sophisticated hardware and signal processing techniques, the technology offers significantly reduced spatial resolution, due to the diffuse nature of light propagation in tissue. fNIRS devices detect changes in the concentration of oxygenated ([HbO]) and deoxygenated ([HbR]) hemoglobin molecules in the blood. fNIRS, like fMRI, is an “indirect” neuroimaging tool in the sense that it monitors hemodynamic responses to neural activations on the basis that neural activations are tightly coupled to vascular processes; a process known as neurovascular coupling. Based on these properties, fNIRS has been shown to have a broad spectrum of uses including studies of vision (20), hearing (22), speech (23), learning (24), emotion (25), and pain (26), and as such, recently has also begun demonstrating its use within the field of PDOC (27–29). Furthermore, as a component of neurovascular coupling relies on end to end asynchronous electrical signaling to drive neural activations, there is growing interest in simultaneous electroencephalography (EEG)-fNIRS—both of which share similar advantageous properties (e.g., portability, inexpensive, and non-invasive) (27).

In this review, we provide a basic overview of fNIRS and its instrumentation [for an in-depth review, please refer to Ref. (30)], followed by discussions of its use within the field of PDOC. We explore the current paradigms used to detect awareness and demonstrate how fNIRS both independently and when simultaneously combined with EEG can accurately monitor changes in neural activity. Next, we explore recent advances to improve the spatial resolution of the signal and methods to advance analysis of the hemodynamic response. Finally, we discuss the potential of fNIRS as a BCI to aid in communication and to improve accuracy of clinical diagnoses.

## Principles and fNIRS Instrumentation

**Figure 1:**
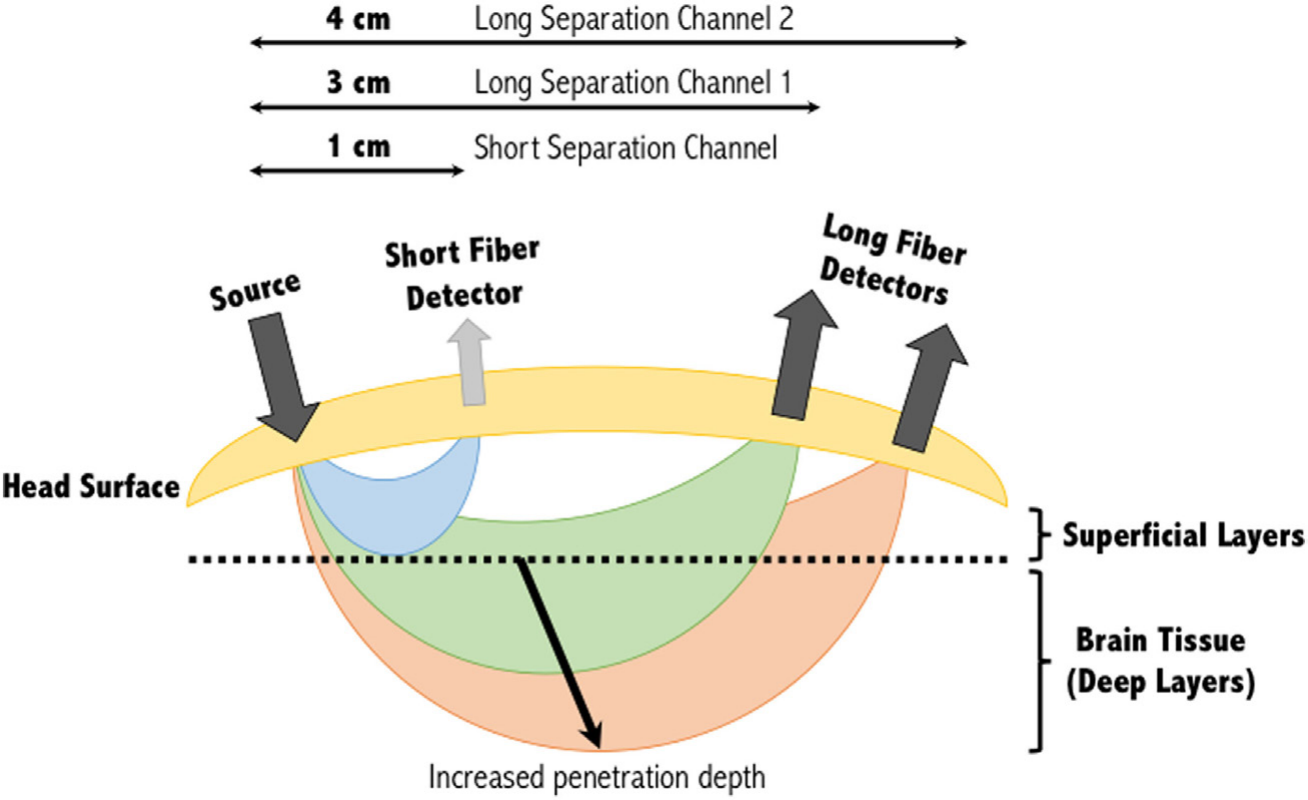
Banana shape profiles of the sampled functional near-infrared spectroscopy signal at multiple source-detector distances. A single source and detector constitute the simplest NIRS channel. Depending on the source-detector separation distance, and the subjects’ skull and scalp thicknesses, the light may or may not sufficiently penetrate the superficial layers to sample the deeper layers. A separation of 3 cm is commonly used, however, increasing this to 4 cm can increase the penetration depth of the light sampled tissues. Short separation channels are located within 1 cm of the source and can provide physiological (noise) data within the superficial layers. This activity can then be regressed from the long separation channel, resulting in a signal corresponding to activity solely within deep brain tissues. Figure adapted from Ref. (34). No permissions were required.

Spectroscopy is based on the study of light signals. In the near-infrared (NIR) range of light, with wavelengths between ~600 and 900 nm, biological tissues are effectively transparent. The low molar absorptivity of lipids and water in this region enables light to effectively penetrate and be maximally absorbed by oxygenated (HbO) and deoxygenated (HbR) hemoglobin (31, 32). These primary light-absorbing compounds in tissue in the NIR range are called chromophores (31). Optical neuroimaging using fNIRS typically requires the use of a set of light-emitting diodes (light sources) on the scalp, and an equal or larger set of detectors, depending on the number of source-detector channels required. NIR light of wavelengths specific to each biological chromophore will be absorbed primarily by that chromophore (HbO, HbR, and cytochrome *c*-oxidase). Scattered light then follows a trajectory back toward the surface of the scalp, in a characteristic “banana” shape, where it is captured and recorded by, for example, photodetectors (Figure 1) (33).

**Figure 2:**
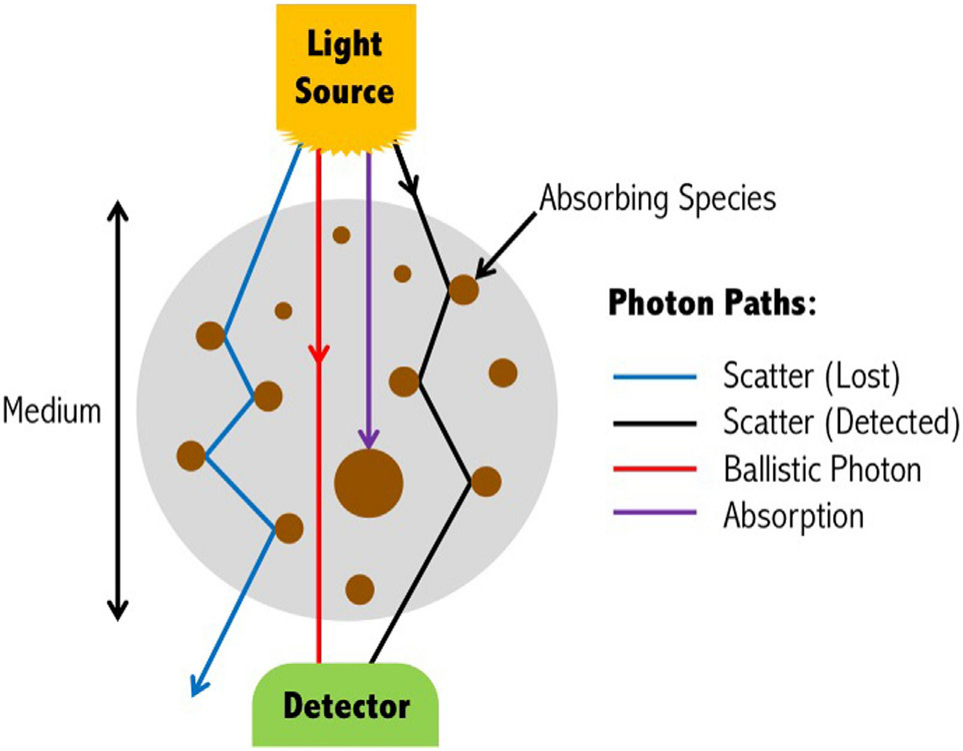
Light propagation paths through a medium. Depending on the wavelength of the emitted light, photons may either be absorbed by the medium, scatter to the extent that they are no longer detectable, scatter and yet be detected, or travel through the scattering medium in a straight-line (ballistic photon). For functional near-infrared spectroscopy devices, ballistic photon paths are highly unlikely to occur due to source and detectors being positioned on the surface of the head, and the light propagating directly into the brain. Figure adapted from Ref. (35). No permissions were required.

*Absorption* and *scattering* are the two main attenuating interactions that take place between light and tissue (Figure 2). As light from a source penetrates through the layers of the head, specific wavelengths will be absorbed by the absorbing (chromophore) components within the different media. The photons that reach the detector on the scalp are primarily those that have scattered within the medium, and therefore have traveled a greater distance than the geometrical (straight-line) distance between the light source and detector. The measured intensity at multiple wavelengths is then used to separate the absorption due to different chromophores. Due to the scattering properties of light on route to the detector, the fNIRS signal has limited spatial resolution of the underlying chromophore concentrations with respect to its location in the head, but contains rich contrast (i.e., a small change in attenuation change will result in a large measured intensity change).

The depth within the skull that can be studied using fNIRS is largely dependent on the inter-optode distance or source-detector separation distance as it is also referred. As a general approximation, the penetration depth achievable is approximately a third to half the source-detector separation distance (21, 36). At greater source-detector separation distances, deeper penetration of light is achieved at the cost of poorly resolved images due to less light being captured by the detector (Figure 1). Diffuse optical tomography can improve this resolution by employing a large number of over-lapping measurements, each generating their own banana-like trajectory. Combining these signals allows a deeper three-dimensional reconstruction of the hemodynamic signals from the brain (37).

Hemodynamic signal integrity can be readily compromised by the effect of superficial layers on the detected signal. These layers of tissue are assumed to have a constant attenuation effect on the light signal; however, there is a slight effect due to extracerebral signal components (38). The attenuating layers in the head include the skin, scalp, skull, cerebrospinal fluid, gray matter and white matter, in addition to the chromophores within the blood. Of these however, the scalp and skull have been shown to be most significant (39). Traditionally, it was assumed that hemodynamic changes in the overlying tissue layers were uncorrelated with the changes in brain function. However, research has shown that the systemic physiological signals from superficial layers can exponentially decay the light from the emitter (40); that is to say that NIRS measurements are inherently most sensitive (have largest magnitude) to tissue nearest the source and detector (40). Major contributors of physiological interference include heartbeat (1–1.5 Hz) (41), respiration (0.2–0.5 Hz) (42), low-frequency oscillations including Mayer waves (~0.1 Hz) (43), and task-related changes in systemic physiology (44).

The mean scalp plus skull thickness in an adult human is typically 10–18 mm [average modeled values of ~7 mm for scalp and ~6 mm for skull as reported by Ref. (39)]. Okada and Delpy showed that increasing the skull thickness from 4 to 10 mm would result in an 80% loss in NIR signal intensity (45). In contrast, Strangman et al. argued that the scalp consistently had a greater influence on NIRS brain sensitivity than skull (39). In addition, they looked at how source-detector separations could overcome this and found that as separations increased above 20 mm (mean sensitivity of 0.06), the effect of the superficial layers became less influential, with near-maximal sensitivity to brain tissue being achieved at or above 45 mm (mean sensitivity of 0.19) (39). Other methods of effectively detecting absorption changes from deep brain tissues while keeping a normal source-detector separation distance (e.g., 45 mm) include the use of independent component analysis (ICA) (46), principle component analysis (47), and model-based analysis such as the general linear model (GLM) (48).

With multiple-distance optodes (i.e., a short separation channel and long separation channel), some groups have shown this method to advance a GLM approach in eliminating superficial effects (49–51). In this approach, short separation detectors that are located in the activation area but have shorter source-detector separation distances (<10 mm) are more sensitive to activity in the superficial layers, whereas the signal received at the long separation detectors are sensitive to both the brain and superficial layers (Figure 1). Regressing out the short separation signal from the long separation signal effectively filters out the superficial component [see (44) for more information about how the data from the short separation channel is regressed from that of the long separation channel]. Other approaches to improve deep tissue spatial resolution with multiple-distance probes include the use of multi-distance probes along with ICA (52), and diffuse optical tomography (53). Alternatively, low processing options to eliminate physiological signals include low-pass filtering (only to eliminate cardiac oscillations) (54) and wavelet filtering (55).

According to a recent investigation by Pfeifer et al., the lack of a standardized signal processing method or guideline for fNIRS data is likely to cause novice users to employ data analysis tools provided by commercial companies (i.e., a “black box”) which are unlikely to take into account the parameters of the study (56) and may increase false positives or false negatives in the final published results (57). Indeed, Pfeifer et al. demonstrated statistical discrepancies between a “black box” signal processing stream, and that of a relatively simple self-implemented signal processing stream that involved motion artifact removal and band-pass filtering of [HbO] and [HbR] data (56). With increasingly widespread use of NIRS devices across biomedical research, the field will clearly benefit from standardization, as adopted in much of fMRI research (e.g., FMRIB Software Library and Statistical Parametric Mapping) (58–63). Furthermore, when using signal processing methods as provided by manufactures, it is paramount that the research team have an advanced understanding of every step to ensure that the data and conclusions are reliable and interpretable.

**Figure 3:**
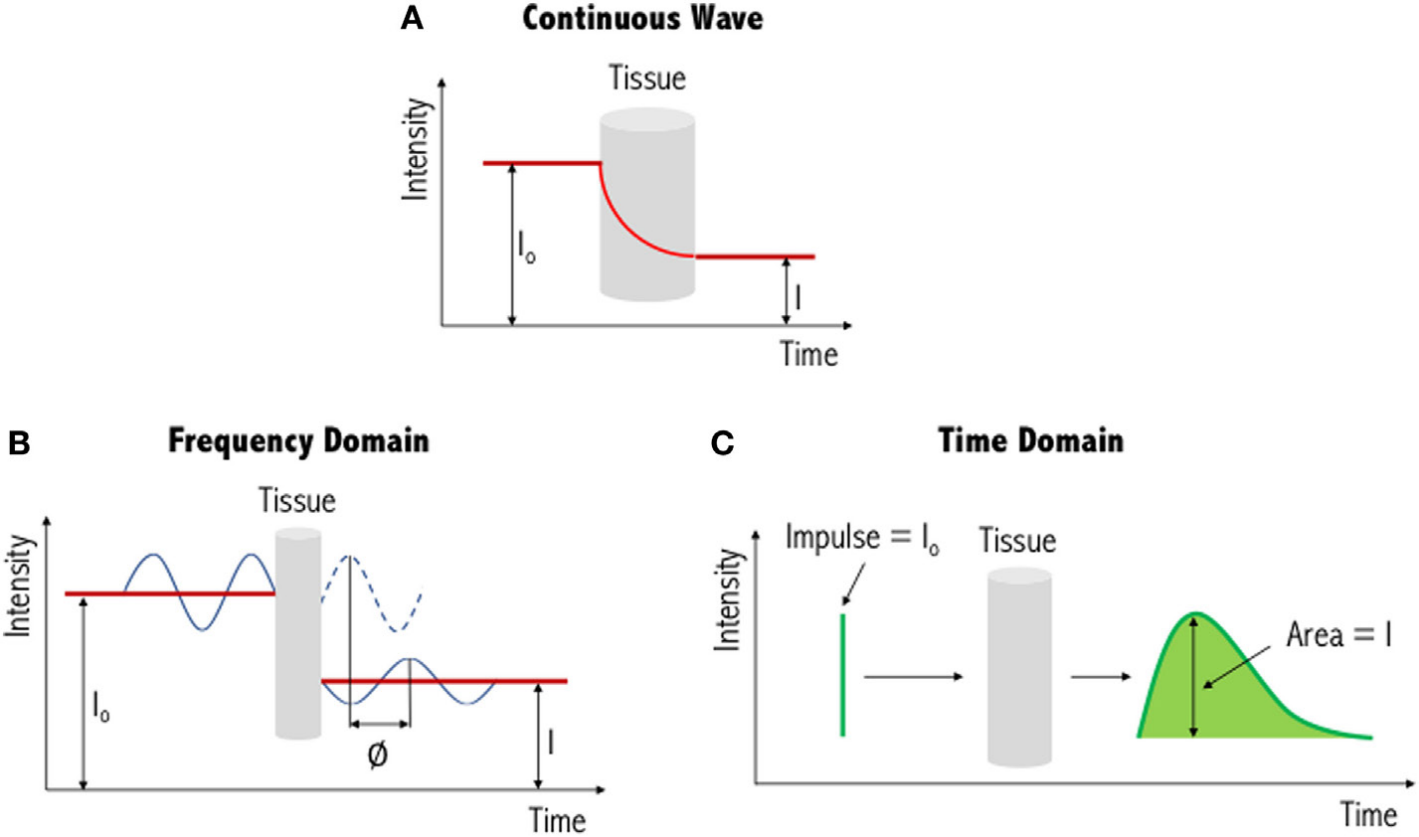
Illustration of three different functional near-infrared spectroscopy techniques. The simplest and most commonly used method is continuous wave near-infrared imaging (top) **(A)**, which measures changes in light intensity having passed through the tissue. Two other methods—frequency domain (bottom left) **(B)** and time domain (bottom right) **(C)**—are variations of this and provide increased information content (see text for further details). I_0_: incident light signal, I: detected light signal and *∅*: phase shift. Figure adapted from Ref. (71). No permissions were required.

The three types of systems that are primarily used for NIR imaging are continuous wave (Figure 3A), frequency domain (Figure 3B) and time domain/resolved (Figure 3C). Of these, continuous wave devices are the most common instruments for measuring the fNIRS signal. These devices emit light at a constant intensity and measure changes in the intensity of the re-emerging (i.e., diffusely reflected) light, having passed through the tissues. To quantify chromophore concentrations from the recorded light intensities requires modeling of the medium through which the light has propagated. The earliest model is the Beer–Lambert law, proposed following work by the French mathematician Bouguer in the 1700s (please refer to discussions and citations in (64)). This type of spectroscopy represents a linear relationship between absorbance and concentration of an absorbing species, and as such has been widely used in colorimetric analysis, with similar principles applied to biological tissue. Biological tissue, such as the brain, is a highly scattering environment. To account for such scattering of light, Delpy et al. developed the modified Beer–Lambert law (65, 66). This has been used widely in continuous wave devices as a means to derive concentration changes of each chromophore (HbO, HbR, and total hemoglobin, HbT). In order to gain absolute concentration values, as opposed to the changes in concentration of each chromophore, other methods of chromophore estimation include spatially resolved spectroscopy (67, 68), time-resolved spectroscopy (69), and phase-resolved spectroscopy (70) systems. The output values of these systems can be seen as approximations as several assumptions are used to determine the optical properties of the tissues (i.e., light scattering and absorption coefficients).

In addition to continuous wave measurements, two other diffuse optical measurements that have been developed include frequency domain and time domain fNIRS. In the former, light sources emit light continuously, like continuous wave-fNIRS; however, the amplitude is modulated at frequencies in the MHz range. The absorption and scattering properties of tissues are then obtained by recording the amplitude decay and phase shift (delay) of the detected signal with respect to the incident beam (43). In time-resolved fNIRS, short (picosecond) incident light pulses are introduced into tissues and as they penetrate through the various layers (i.e., skin, skull, cerebrospinal fluid, and brain) the signal is broadened and attenuated. As the photons leave the tissue, the recorded temporal distribution by the time domain system, and the shape of this distribution, provides information about tissue absorption and scattering. Advantages and disadvantages of these three systems are summarized in Table 1. From Table 1, we can see that while continuous wave fNIRS devices offer a cheap and portable method of rapidly capturing brain hemodynamic activity, their simplicity limits the spatial resolution and the penetration depth that can be achieved in comparison with frequency- and time-domain fNIRS systems.

**Table 1:** Advantages and disadvantages of the three commonly used functional near-infrared spectroscopy techniques.

Measurement type	Advantages	Disadvantages	Reference
Continuous wave	▪ High sampling rate ▪ Can be miniaturized—ease in portability ▪ Simple to use ▪ Low cost	▪ Low penetration depth—increased sensitivity to superficial layers ▪ Difficult to separate absorption and scattering	(71, 72)

Frequency domain	▪ High sampling rate ▪ Relatively accurate separation of absorption and scattering	▪ Moderate penetration depth—sensitive to superficial layers	(73, 74)

Time domain	▪ High spatial resolution ▪ High penetration depth—mean time-of-flight and variance values can separate brain tissue from superficial layers ▪ Most accurate separation of absorption and scattering	▪ Low sampling rate—greater loss of photons ▪ Instrument size/weight is larger ▪ Stabilization/cooling required ▪ Costlier system as most advanced ▪ Can be more susceptible to noise—can impact the usefulness of studying variance values	(75, 76)

*Table adapted from Ref. (21)*.

## Motor Imagery Paradigms with fNIRS

Motor imagery is the imagined movement of the body while keeping the muscles still. Motor imagery tasks can provide proxies of command-following for those patients who may be aware but unable to produce purposeful overt behaviors. However, motor imagery-BOLD activation is not always detectable in all participants; indeed, Fernández-Espejo et al. found no appropriate activation in 20% of healthy participants in one study (77).

A variety of motor imagery paradigms have been examined for use with fNIRS (Table 2), the majority of which require activation of the hand and foot areas of the cortical homunculus. Motor imagery tasks can be divided into visual and kinesthetic tasks. In the former, the participant visualizes the movement while in the latter the participant imagines the feelings and sensations produced by the movement. Kinesthetic motor imagery is more often used as it has been shown to recruit more of the cortical motor system (78). Coyle et al. used a continuous wave-fNIRS system to demonstrate that when three healthy participants imagined squeezing a ball, their [HbO] increased reliably above that from rest in the C3 and C4 regions of the motor cortex (based on the EEG international 10-20 system), regions predominantly associated with hand movements (79). Interestingly, after averaging each participants’ data over 20 trials, hemodynamics following motor imagery activation could be prominently distinguished by eye from that of baseline prior to signal processing. Although this may indicate that such experimental paradigms can generate profound neuroactivational changes, it is important to note that their findings were based off a small cohort of three participants. Nevertheless, the authors were further able to show that, by solely studying HbO changes, motor imagery could be used to correctly classify a user’s intent ~80% of the time. Other types of motor imagery paradigms that have established significant hemodynamic signal changes with fNIRS include tennis arm-swinging motion (80) and a finger tapping sequence (81).

**Table 2:** Comprehensive list of functional near-infrared spectroscopy motor imagery studies, including those that have also been applied within a brain–computer interface (BCI) setting.

	Measurement type	Channel density	Wavelengths (nm)	Reference
Motor imagery	Time domain	4	760, 830	(80, 82)
	Continuous wave	18	760, 850	(83)
	Continuous wave	24	695, 830	(84)
	Continuous wave	24	695, 830	(85)
	Continuous wave	48	695, 830	(86)

Motor imagery-BCI	Time domain	4	760, 830	(87)^a^
	Frequency domain	8	690, 830	(88)
	Continuous wave	2	760, 880	(79)
	Continuous wave	4	760, 870	(89, 90)
	Continuous wave	16	760, 850	(27)^a^
	Continuous wave	20	780, 805, 830	(91)
	Continuous wave	24	695, 830	(92)
	Continuous wave	24	695, 830	(93)
	Continuous wave	24	695, 830	(92)
	Continuous wave	24	695, 830	(94)
	Continuous wave	24	695, 830	(95, 96)
	Continuous wave	24	760, 830	(97)
	Continuous wave	24	760, 850	(98)
	Continuous wave	24	780, 805, 830	(99)
	Continuous wave	31, 14	780, 805, 830	(100)
	Continuous wave	34	760, 830	(101)
	Continuous wave	40	760, 830	(102)
	Continuous wave	45	780, 805, 830	(103)
	Continuous wave	48	780, 805, 830	(104)
	Continuous wave	50	780, 805, 830	(105)
	Continuous wave	50	780, 805, 830	(106, 107)
	Continuous wave	52	695, 830	(108)
	Continuous wave	52	780, 830	(109)
	Unknown	1	700, 880	(110)
	Unknown	24	740, 808, 850	(111)

*Included are the types of measurements being recorded, channel density, and the types of wavelengths being operated*.

*^a^Indicates studies that have been conducted on patients with prolonged disorder of consciousness or locked-in-syndrome (please refer to Table 3)*.

Aside from these, of popular interest with fNIRS is the ability to differentiate activations from left- and right-hand movements whether that be tapping, gripping or flexing of the wrist. Sitaram et al. reported that fNIRS recordings of motor imagery for left- and right-hand tapping were similar to motor execution recordings, but smaller in magnitude (91). Nevertheless, from the data it was clear that the hemodynamic responses for left-hand and right-hand motor imagery had distinct patterns that could be used by a classifier to discriminate between the two classes. As such, the researchers of this study were able to achieve approximately 89% accuracy using their classifier, with similar results being achieved by others (87% accuracy achieved when distinguishing between imagined right-wrist and left-wrist flexion) (91, 101).

To add to the hand tapping motor imagery paradigm, recently there has been significant interest in separating left and right foot tapping’s using fNIRS. When using a four-class motor imagery paradigm (left/right foot/hand) in a BCI setting, Batula et al. achieved an average classification accuracy of approximately 46% over three participants (chance = 25%; two participants had a classification accuracy over 50%) (93). Nevertheless, the authors suggested that improved performance could be achieved by utilizing more informative features or classifiers through a more detailed inspection of the activation patterns, or a better selection of motor tasks. However, from their confusion matrix, it can be seen that right foot was most frequently misclassified. This is not surprising as distinguishing between left and right foot using fNIRS is challenging as the foot motor areas are near or within the longitudinal fissure between brain hemispheres (112). Nevertheless, improvements to classification accuracies could be achieved by using a single “feet” or leg motor imagery task (113), or by providing feedback training to strengthen the participants motor imagery abilities (114).

Many of the NIRS systems currently employed in motor imagery research are continuous wave (Table 2), and so require extensive montage (source and detector layout) development and data processing. However, time domain-NIRS devices have the potential to enhance depth sensitivity as they record the arrival times of individual photons to build a distribution of times of flight (115, 116). Early work by Abdalmalak et al. assessed the feasibility of time domain fNIRS to detect brain activity during motor imagery (80). Seven participants performed tennis-playing imagery of which four showed prominent activity in either the PMC alone or PMC and SMA, as detected by fMRI. During the task, increases in blood flow and volume in the PMC and/or SMA led to an increase in light absorption, and thus a decrease in the number of photons, *N*, reaching the detector and their mean time-of-flight, <*t*>. These changes in *N* and <*t*> precisely occurred during the onset of motor imagery and not during rest for the four participants that likewise showed fMRI activity. On a small scale, this study demonstrated good agreement between both imaging modalities, strengthening the argument for the use of fNIRS in motor imagery. However, in three of the seven healthy participants, who were demonstrably aware, no activity was detected by either imaging modality. While no method will be perfectly sensitive (77), it is clear that considerably greater levels of sensitivity are required before this method may be used clinically. Therefore, the same authors tested 15 healthy participants with the same tennis-playing imagery task and instead evaluated the mean and variance, which have greater depth sensitivity, and report sensitivity values between 86 and 93% in the SMA and PMC, the highest being for <*t*> as the data are less influenced by noise (82). Furthermore, of the 15 participants that took part in the study, 93% generated responses that were detectable by fMRI and 87% by fNIRS, a considerable improvement over their earlier work (80) and a clear demonstration of the power of advances in physical and computational methods to improve detection of clinically meaningful information from fNIRS signals. These promising results also confirm that time domain fNIRS is an alternative means of reducing scalp contamination and for enhancing the sensitivity to brain activity, and thus may be a well-suited tool for use on patients with PDOC. To the best of our knowledge, time domain fNIRS data have not yet been reported in patients with PDOC.

Research in patients with PDOC has, however, been accomplished using other fNIRS devices. Molteni et al. detected residual functional activity in two minimally conscious state patients using a commercially available NIRS device (although undefined in the manuscript) and a protocol that involved somatosensory, passive movement, and active movement stimulations (28). While somatosensory stimulation (using a vibrating pillow) elicited a weak response over the somatosensory cortex, passive movement stimulation (hand movement with the assistance of the experimenter) generated clearer hemodynamic responses (increase in HbO, decrease in HbR). Active movement tasks (self-performed hand opening and closing) generated the weakest hemodynamic response in the hand region of M1 in both patients; however, this was expected as the patients were unable to move their hands autonomously and showed no signs of engagement with the task. Furthermore, their T1-weighted MRI brain scans indicated the presence of severe atrophy that could have allowed for fluid accumulation. An excess in cerebrospinal fluid would have increased the attenuation of the NIRS signal (see earlier discussions) thereby reducing the chance of a measurable response to the task. Overall, as a primary study, the authors were able to show that residual brain activity can be detected in patients with PDOC using fNIRS and favors the use of motor imagery as a means of overcoming the need for patients to execute movements, which may not always be possible.

In a study by Kempny et al., 16 patients (11 in a minimally conscious state and 5 in a vegetative state) performed a kinesthetic motor imagery task of squeezing a ball with their right-hand whilst being evaluated with continuous wave-fNIRS (27). In addition, healthy participants were asked to physically perform and kinesthetically imagine the same task in order to obtain patterns that could be used to validate responses in patients with PDOC. A typical fNIRS response to movement and motor imagery is an increase in the [HbO] accompanied by a less pronounced decrease in the [HbR] (117, 118). However, the groups in this study exhibited two types of responses during motor imagery; the typical responses and an inverted response (decrease in [HbO] and an increase in [HbR]). Furthermore, minimally conscious patients, in comparison with those in a vegetative state, more often exhibited a hemodynamic response that was similar to that of healthy participants. Fluctuations in hemodynamic patterns have been shown to depend on the location of the probe and the difficulty of the task (106), highlighting the importance of normative data from healthy individuals against which to compare a given patient’s response. Kempny et al. further identified that the greatest reduction in [HbO] was found on the right hemisphere of the head across all three groups during motor imagery (27). Regions of hemodynamic activation were in line with previous studies (118, 119), with greater activation observed on the ipsilateral side [see (95) for similar results]. While this may seem unusual as one would expect primarily activation of the contralateral areas during hand motor imagery, Batula et al. demonstrated that this is not always the case, in particular when the left-hand is involved, which generated a more bilateral activational pattern during motor imagery (95), a pattern confirmed by fMRI (120, 121).

The above studies demonstrate the feasibility of fNIRS in the field of PDOC. However, there is much to do to ensure that the signals measured are sufficiently reliable and interpretable for use in clinical contexts. Below we suggest one potential means of achieving that goal.

## Simultaneous EEG-fNIRS

One means of improving the sensitivity of fNIRS, while maintaining portability, is to combine it with simultaneously acquired EEG. During neural activity, glucose and oxygen are rapidly consumed from the local capillary bed. This reduction in metabolites stimulates the brain to increase local cerebral blood flow and cerebral blood volume. A number of models have been proposed that both physiologically and mathematically demonstrate the association between electrical and hemodynamic responses (122, 123), strengthening the existence of neurovascular coupling. This has led some to even study the phenomenon at the bedside, highlighting the delay in the vascular response in comparison to neural activation during stimulus onset (124). As such, this reinforces the argument for the simultaneous use of fNIRS along with an electrophysiological method to better understand the underlying brain activity in patients with PDOC.

During neural activity, summation of ionic fluxes across large numbers of synchronously activated neurons (dipoles) can cause changes in electric fields that can be measured directly using EEG with high temporal resolution (millisecond timeframe). EEG passively measures scalp surface potentials and has been widely explored for identifying covertly aware patients (125) and monitoring rehabilitation success (126) within the field of PDOC [for a detailed overview of EEG, see Ref. (127)]. EEG shares many advantageous properties with fNIRS including its portability, low cost and non-invasiveness. Nevertheless, EEG is prone to blink artifacts, which can be readily eliminated with the use of computational tools such as ICA (128). Alternatively, and depending on the outcomes of the study, participants could close their eyes; however, this can corrupt task-related signatures with physiological noise (129, 130). For example, Verleger reported that refraining from blinking lowered the amplitude of the characteristic P3 peak (a positive-going component of an EEG signal) during an auditory task (130). In addition to ocular movements, the spatial resolution of EEG can be relatively poor. This is due to the spatial smearing of the EEG signal, through a process known as volume conduction (131), as each dipole exerts influence in nearly all directions and not just on the scalp immediately above the dipole. Computational tools, such as the use of spatial filters [e.g., the surface Laplacian (132)], offer a solution to improve the spatial resolution of the dataset. However, this can be further enhanced when EEG is used simultaneously with fNIRS, because the improved spatial resolution offered by fNIRS can provide some degree of information regarding the active source’s location, thus complementing EEG findings.

Demonstrating the link between brain hemodynamics and electrophysiology, Zama and Shimada reported a strong correlation in healthy individuals between the magnitudes of the change in HbO in contralateral PMC and the EEG-detected readiness potential approximately 1,000 ms before movement onset (133). As both electrical and optical tools measure different aspects of brain activity and do not interfere with one another, there is also potential that simultaneously acquired EEG and NIRS data will contain complementary information about brain activity and/or neurovascular function that cannot be observed when using these systems independently.

**Figure 4:**
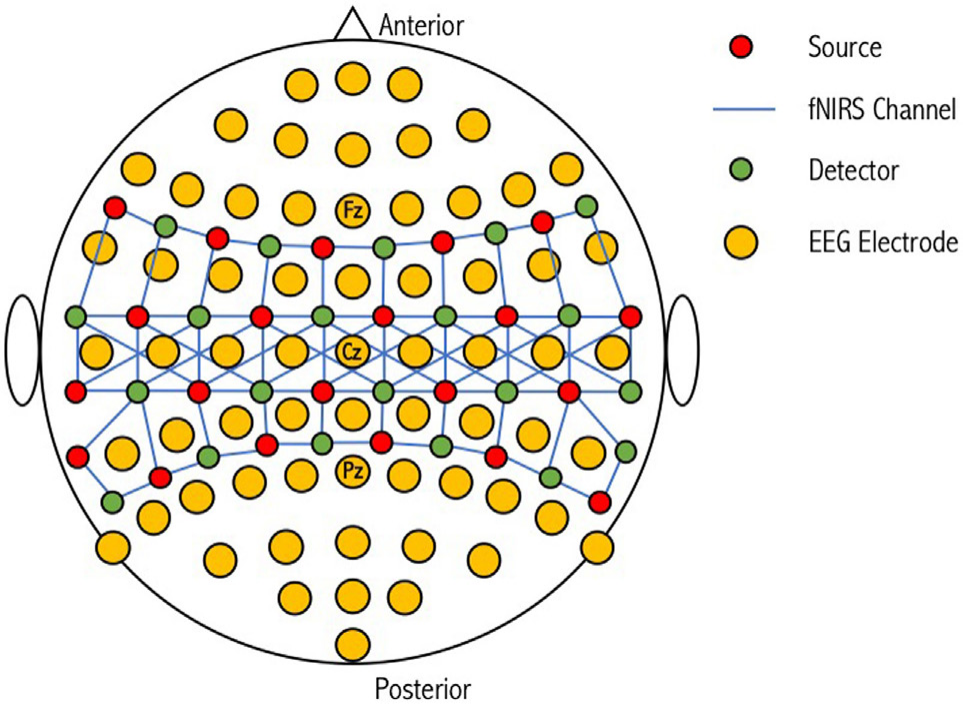
Schematic representation of a combined optode-electrode head probe. The electroencephalography (EEG) international 10-20 positioning system is used to form the base for 64 EEG electrodes. NIRS sources and detectors are then placed in close proximity to these electrodes to form corresponding channels of different lengths. An increase in the number of sources and detectors used results in an increase in channel complexity and an overall improvement in the resolution of the sampled tissue. Figure adapted from Ref. (100). No permissions were required.

While EEG electrode positioning is commonly based on the International 10-20 and 10-10 positioning systems, there is no accepted standard for NIRS optode placement on the scalp, although attempts have been made to match the International 10-20 system used in EEG (134). Low electrical impedance is desired for high-quality EEG because neural signals are small (microvolts) relative to background noise (135). This is achieved by using electrode pastes and gels. Improving optical coupling for fNIRS devices is achieved by increasing the efficiency of light transmission between the optode and the head. Light can be lost both at the source and the detector if air gaps are introduced (136); however, this loss can be minimized if optodes are positioned in direct contact with the scalp surface. During simultaneous use, electrode gels and pastes on the scalp for EEG recording can negatively impact the transmission of light for fNIRS. Giacometti and Diamond designed an EEG-fNIRS head probe that linked NIRS channels through EEG electrodes (137). Their head probe can stretch to fit a wide range of head sizes and account for head shape variability while maintaining contact pressure on the scalp. Furthermore, relative to other commercial products, their head probe was found to have improved accuracy (i.e., the sensor is placed on the location where it corresponds with the EEG 10-10 standardized system, 83.2%) and precision (i.e., the sensor is placed on the same location on a particular head every time, 39.5%). This design is, however, limited by the number of combined optode-electrodes that can be positioned on the scalp (Figure 4). Cooper et al. designed an integrated opto-electrode probe that housed both an EEG electrode and an optical fiber bundle (138). With this device, they observed a hemodynamic response during a finger-to-thumb opposition task alongside an EEG readiness potential. Open-source and commercial hardware is therefore available to promote research into simultaneous EEG-fNIRS for detection of covert consciousness and cognition.

### Simultaneous EEG-fNIRS in BCI Applications

The majority of EEG-fNIRS studies have been within the field of BCIs. BCIs are used for several applications including spelling devices, environmental control, navigation in virtual reality, simple computer games, cursor control applications, and control of prostheses and robotic arms (139–142). The most commonly studied signals in BCI are those of EEG. Neuronal oscillations observed in EEG are categorized into five specific frequency bands: delta, <4 Hz; theta, 4–7 Hz; alpha, 8–12 Hz [also known as mu activity when recorded from sensorimotor areas (143)]; beta, 12–30 Hz, and gamma >30 Hz. A decrease in oscillatory activity in a specific frequency band is known as an event-related desynchronization, whereas a corresponding increase is called an event-related synchronization. Event-related desynchronization/synchronization patterns are produced in motor imagery as a result of mu and beta frequency band activity within the EEG signal (144). There have been several applications of EEG-based BCI in the field of PDOC, rehabilitation, and for other conditions resulting from a traumatic brain injury or motor impairing disease [for a detailed list of references, please refer to Ref. (145)].

In fNIRS BCI, features for classification are mostly extracted from hemodynamic signals (HbO, HbR, and HbT), such as peak amplitude, mean value, variance, slope, skewness, kurtosis, wavelet transform, and those from genetic algorithms (146). A combination of these then makes a training set that are used to train a classifier (supervised learning) before applying a test data set for it to detect brain-signal patterns (147). For a two-class problem (i.e., left- and right-hand imagery) support vector machines (SVM) are greatly favored, as they attempt to maximize the distance between the separating hyperplane and the nearest training points—or so-called support vectors (146). Other classifiers include linear discriminant analysis (LDA), artificial neural networks, and hidden Markov models (148). The classified signals are then sent to an external device to generate the desired response. In neurofeedback tasks, the response is a display of the accuracy of the users’ intent, based on their brain activity allowing self-regulation of brain functions.

There has been a growing interest for the use of fNIRS BCI as a communications device for patients in a locked-in state. Locked-in-syndrome (LIS) is a condition in which patients are aware but have limited or no means to move or communicate (149). As a result, they share similar challenges as those diagnosed with PDOC (i.e., patients in an early minimally conscious state are clinically aware but can lack the mobility to respond to commands). Naito et al. examined the ability of patients with LIS to communicate “yes” or “no” answers to several questions through either performing a mental calculation or by mentally singing (assigned “yes”), or by staying mentally relaxed (assigned “no”) (150). Frontal lobe activity was measured using a self-devised continuous wave-NIRS device, and the extracted amplitude and phase data were used in a discriminant analysis to classify the patients’ response. Of the 17 patients with LIS participating in the study, only 40% (i.e., *n* = 7) had significantly differentiating responses. From these seven patients, the average rate of correct detection of their intention was 80%. The limited applicability of this BCI in patients with LIS can be accounted for by the lack of specificity of optode placement, as well as the partial filtering applied to the dataset. A low pass filter with a cutoff frequency of 0.1 Hz was applied, and hence this may not have completely eliminated Mayer waves, which are known to readily corrupt the NIRS signal when sampled from the superficial layers (110).

Functional near-infrared spectroscopy is a relatively novel technique in the field of motor imagery-based BCIs, with EEG still viewed by many as the gold standard. Table 2 displays a list of several fNIRS motor imagery studies, with the majority being extended for use in BCI research. On the whole, there is widespread use of continuous wave-fNIRS devices as these low-cost instruments are relatively easy to set up and use. The number of channels used for a study ranges between 1 and 52; however, this is dependent on the number of sources and detectors available for a particular device and the region of the head it is required to cover. Furthermore, those studies using less than 24 channels have generally been experimental for example to test novel probe or sensor designs. With respect to the type of wavelengths used, these range between 695 and 850 nm, all of which are within the acceptable limits to measure changes in the biological chromophores. More complex and costlier instruments such as time domain (see section 3) and frequency domain fNIRS have had far less use within this research community. Koo et al. demonstrated the reliability of a hybrid (fNIRS and EEG) self-paced motor imagery-based BCI using a frequency domain NIRS system (88). Here, self-paced motor imagery is where the onset of motor imagery is not known, and neither are the brain signals corresponding to the detected motor imagery (in cue-based motor imagery, the start or cue of the motor imagery is known, hence a BCI system can recognize the motor imagery from the participants brain signals). While a frequency domain system was used for the study, which aided in the hybrid BCI achieving true positive rates of 88% (i.e., the BCI well recognized the intentions of the participants), it was clear that no phase data was extracted and analyzed, and thus the instrument was analyzed as if it were a continuous wave system. The majority of work using frequency domain systems has not yet extended beyond motor execution studies (i.e., tapping tasks), with those using the device either evaluating both time-domain and frequency domain (phase) parameters (151), or fast optical signals (152, 153) [see Ref. (73, 154) for more information regarding fast optical signals and event-related optical signals].

Several research groups have opted for a hybrid BCI approach whereby NIRS features are used to support and complement EEG-based BCI. Fazli et al. conducted motor execution and EEG-based, visual feedback controlled motor imagery tasks on 14 healthy right-handed volunteers, requiring them to perform left- and right-hand gripping (98). Twenty-four fNIRS channels (8 sources and 16 detectors) and 37 EEG electrodes were used for data acquisition. The NIRS data were then low pass filtered (0.2 Hz) and baseline corrected before using the modified Beer–Lambert law to calculate concentration changes of hemoglobin. Time-averaged concentration changes (HbO and HbR), using a sliding window, were then used as features for LDA classification. EEG band-pass filtered coefficients in the alpha and beta bands were spatially filtered using a method known as common spatial patterns (155) before the LDA classifier was computed. The LDA results from EEG, [HbO], [HbR], and combinations of all three were fed into a meta-classifier before testing. On average for motor imagery, combining EEG with either [HbO], [HbR], or both, had a classification accuracy of approximately 82%, statistically significant than the accuracies of the individual methods (EEG: ~78%, HbO: ~72%, HbR: 65%). However, it has recently been shown that both age and feedback can affect motor imagery patterns during simultaneous EEG-fNIRS data acquisition (83).

Use of a large number of fNIRS channels and/or EEG electrodes during simultaneous EEG-fNIRS acquisition can in some cases be suboptimal due to the high dimensionality of the data and the associated computational costs during classification. One approach to dimensionality reduction is the widely used EEG spatial filtering method of common spatial patterns. Importantly, applying this method to fNIRS data will align the EEG and fNIRS processing streams, allowing for more meaningful comparisons across modalities while improving classification accuracies when used simultaneously in BCI applications. The common spatial patterns method in multichannel EEG (155) efficiently reduces the number of features for classification to those that have highly discriminative properties between tasks. Conversely, in fNIRS, the majority of published studies have employed channel-wise HbO and HbR as features for classification (89, 102). This high feature dimension space requires more trials and a longer training time to train the classifier—first introduced by Bellman as the “curse of dimensionality” (156). Furthermore, a high feature dimension may increase the complexity and instability of the classifier. Zhang et al. evaluated a common spatial patterns algorithm on multichannel fNIRS data and found significant improvement in classification accuracy in both motor execution and motor imagery tasks relative to a conventional high dimension feature space (average accuracy with 180 feature dimensions—54%, average accuracy with 18 feature dimensions derived from common spatial patterns—74%) (111). A benefit of high density EEG is that the whole head coverage allows the researcher to confirm the physiological plausibility of the spatial pattern maps associated with each task—i.e., is the activity restricted to electrodes over contralateral sensorimotor cortices for hand imagery? The fNIRS probes used in the work by Zhang et al. were positioned over the motor cortex as this region is primarily activated during a hand tapping task (111). While focusing on a small area of the scalp is beneficial from the perspective of statistical multiple-comparisons and data dimensionality, it is not possible to ensure that the recorded hemodynamic changes are physiologically plausible, or whether they reflect a general amplification in blood flow across the entire brain. Therefore, future fNIRS research could extend toward greater scalp coverage so as to minimize the research gap and align the common spatial patterns methods of fNIRS with those of EEG, while aiming to balance the benefits against the increased preparation time and reduced portability and comfort of whole-head systems.

Recently, there has been interest into the use of a “few-channel” approach for BCIs, based on previous research demonstrating focal neural activation in specific motor tasks. Ge et al. demonstrated the accuracy of a few-channel BCI using EEG-fNIRS on participants conducting a left- and right-hand gripping motor imagery task (100). To validate which few-channels to use for the BCI (i.e., feature extraction and classification steps), the initial paradigm was performed simultaneously using a 64-channel EEG electrode set and 52-channel fNIRS set. Of these 52-channels however, 31 (11 detectors and 11 sources) were placed over the sensorimotor cortices (C3-Cz-C4 in 10-20 nomenclature). From the 64 EEG and 31 fNIRS channels, electrodes at positions C3, Cz, and C4 and 14 fNIRS channels (6 sources and 6 detectors) centered around C3 and C4, were used for the few-channel EEG-fNIRS BCI, as these showed distinct neural activity during both left and right motor imagery tasks [see Figure 3 in Ref. (100) for further information on the montage layout]. Following feature extraction, fusion of both EEG and fNIRS datasets, and classification using SVM, the researchers were able to demonstrate that few-channel EEG-fNIRS had a significantly higher classification accuracy for 11 out of 12 participants than either of the individual modalities (average classification accuracies: EEG—75%, fNIRS—57%, EEG-fNIRS—81%) (100).

In the validation step, source analysis for both the 64 EEG and 31 fNIRS channels was performed to localize the neural signals during the motor imagery task. Source analysis effectively attempts to solve the question of what brain tissues/areas are being probed by a given measurement. In EEG, source analysis involves estimating solutions to the ill-posed inverse problem. Due to the effects of volume conduction, sources (i.e., dipoles) all over the brain may contribute to a measured signal at the scalp. However, an infinite number of source configurations (i.e., radial and tangential) may generate a particular pattern of voltage at the scalp (127). Source analysis thus requires estimating the final surface voltage pattern and then working backward to determine which neural sources generated that voltage pattern. As a result of this, the inverse problem can be seen as a NP-hard (non-deterministic polynomial-time hard) problem, where no absolute answer is available. Nevertheless, several methods are available, based on certain assumptions, to obtain approximate solutions (157, 158). In fNIRS, the question for source analysis becomes more specific as we aim to understand the depth penetration of the instrument. Light propagation through scattering media, such as the head (heterogeneous structure) is inherently complex and as such mathematical models of this process (radiative transport equation and its diffusion equation) are difficult to solve analytically (74). Estimations can however be made by solving the diffusion equation for optically homogenous tissues with infinite, semi-infinite, or slab boundary conditions (159, 160). Two types of numerical approaches can also be used to gain information about sensitivity and penetration depth in complex tissue: (1) approaches based on finite element and finite difference analysis or (2) Monte Carlo simulations of photon propagation through the tissue. The latter was used by Strangman et al. to highlight that an increase in source-detector separation increased sensitives of higher level gray matter samples, however at the cost of exponentially decaying sensitivity in-depth penetration (161). Returning to the study of Ge et al., standardized low-resolution electrical tomographic analysis (158) was used to compute an inverse solution for the EEG motor imagery data, whereas digitized points and topographical maps of the changes in HbO, superimposed onto the surface of a standard three-dimensional head model, were used for the fNIRS data (100).

These methodological advances have ultimately aided efforts to improve BCI communication in patients with LIS at the bedside (162, 163). Most recently, Chaudhary et al. demonstrated that patients with LIS could be trained (using feedback) to directly communicate through their hemodynamic signatures “yes” and “no” answers to a number of individually tailored personal questions (163). Patients were specifically asked to think (not imagine) “yes” or “no” when answering the auditorily presented questions while data were recorded from frontocentral brain regions. In the training period, questions with known “yes” or “no” answers were presented. However, to add to the complexity, the trained classifier was then tested using open questions (e.g., quality of life questions: “You have back pain”) to which only the patient could answer. Findings demonstrated that when most patients were instructed to answer “yes” there was an increase in oxygenation that was not followed by a decrease when providing a negative response. As such, the mean relative change in [HbO] across each of the channels was used as a feature to train the SVM classifier through a fivefold cross-validation procedure. Overall, in three quarters of patients it was found that the correct response rate for feedback and open question sessions exceeded 75%. This study marks a huge leap in the capabilities of fNIRS BCI, especially when compared to the study a decade ago by Naito et al. (150). Furthermore, in many cases, it sets the stage for the use of simultaneous EEG-fNIRS BCI in patients with PDOC. Nevertheless, caution should be taken since these results were based off a small cohort of four patients. Additionally, the underlying neurocognitive mechanism is unclear, as responses were not detected via a proxy mental action (i.e., in motor imagery) but by apparent processing of the “correctness” of the statements—i.e., that they were indeed experiencing back pain, rather than that they were performing a mental behavior to signal that they were experiencing back pain. The lack of a clear neurocognitive model may impede its utility in a wider patient group.

This school of thought is opposed to the more widely used method of using proxy behaviors for communication—e.g., imagining playing tennis to answer “yes.” This approach importantly does not rely on unclear models of neurocognitive processing but makes use of a clear signal of volitional command-following. However, command-following places higher cognitive demands on the communicator as they must map the appropriate response onto an arbitrary behavior and produce that behavior. Conversely, the approach of Chaudhary et al. (163) assumes that the communicator’s passive experience of the correctness of the statement is sufficient to provide the communicative output and is therefore a potentially more functional method for patients with limited cognitive resources as a result of brain injury.

In a recent comparison to the continuous wave device used by Chaudhary et al. (163), which is prone to be increasingly sensitive to light absorption in superficial layers and thus less reliable at detecting brain activity, Abdalmalak et al. (87) tested their previously designed four-channel time-resolved fNIRS system (80, 82), which enhances depth sensitivity by discriminating between early and late arriving photons (see previous discussions between continuous wave and time domain systems). With this technique, they detected motor imagery (imagining playing tennis) from a patient who was diagnosed with an acute form of locked-in-state. Furthermore, by using motor imagery as a proxy for communication and by analyzing the mean time-of-flight signals (converted to HbO and HbR and classified using SVM), they detected yes/no responses to a series of questions addressed to the same patient. The accuracy of the answers was confirmed by the patient’s residual eye-movement communication channel. While this method has the potential to be translated to patients with PDOC, postinjury functional reorganization of the brain may affect the choice of probe placement, and ischemia or hematoma can impede scattering and absorption of light. As such, structural imaging data would contribute significantly to increasing the accuracy of fNIRS BCI methods. Furthermore, it is necessary to take into account any medications or sedatives used by the patient, as some are known to cause hemodynamic fluctuations that could be misinterpreted as being task-related (164).

## From Communication to Advances in Rehabilitation

Improving diagnostic accuracy and establishing a means of communication between clinicians and patients has been a significant goal of the field of PDOC over the last two decades. However, there has also been significant research into potential therapies and treatments via pharmacological interventions and neuromodulation techniques, such as deep brain stimulation and spinal cord stimulation (165–167). fNIRS provides a non-invasive means of quantifying the neurophysiological and neurocognitive impact of these approaches.

Unlike deep brain stimulation where an electrode is implanted directly within the brain rupturing the safety of the blood–brain barrier to external pathogens, in spinal cord stimulation the electrode is implanted in the epidural space to stimulate the ascending transmission pathways and regulate the awareness circuit (e.g., the mesocircuit) (168, 169). This method has been applied to PDOC with some promising effects. Kanno et al. reported that 54% of patients (109 out of 201) in a vegetative state began demonstrating purposeful behaviors (170), whereas Yamamoto et al. reported 70% of patients (7 out of 10) recovered from the minimally conscious state (i.e., demonstrated functional interactive communication and/or functional use of two different objects) following spinal cord stimulation use (166). Spinal cord stimulation is known to enhance cerebral blood flow and increase cerebral glucose metabolism (166, 168, 171), stimulate neurotransmitter and neuromodulator release (171, 172), and excite nerve conduction and electrical activity within regions of the brain (168, 171). This multitude of effects may ultimately come together to enhance the recovery process of such patients.

Assessing brain responses during spinal cord stimulation in patients in a minimally conscious state has previously been achieved using EEG. Previous studies have shown significantly altered relative power and synchronization in the delta (1–4 Hz) and gamma (30–45 Hz) bands in the frontal areas following spinal cord stimulation (173), with gamma activity in the frontal cortex causing transient global effects (widespread connectivity and network alterations) and long-lasting local effects (local connectivity alternations that persist beyond stimulation) (174). The drawback with EEG, however, is that brain responses during spinal cord stimulation cannot be measured in real-time due to interference from the stimulator’s electrical field. fNIRS on the other hand is not limited by this issue.

Using an eight-channel fNIRS device (device type not specified in the manuscript), Zhang et al. provided insights into the mechanisms of spinal cord stimulation for PDOC, in addition to quantifying the neuromodulation effects of different stimulation parameters (29). In the prefrontal cortices of eight patients with PDOC, the researchers found a characteristic profile of an increase in [HbT] when stimulation was switched on, followed by a gradual return to baseline after stimulation was switched off. No such meaningful profile was observed in the occipital cortex. Furthermore, in the prefrontal cortex, both patients in the minimally conscious state showed significant increases in HbT across blocks, while such a profile was only present in two out of the six patients in the vegetative state. These results hint that it may be possible to partially increase cerebral blood flow in patients with PDOC via spinal cord stimulation, and that fNIRS can be used as a real-time monitor of these physiological consequences. Continued use of fNIRS alongside explorative therapies for PDOC has potential to guide clinicians in tailoring stimulation protocols to optimize desired physiological responses and ultimately increase the success of rehabilitation efforts.

## Conclusions and Future Perspectives

Functional near-infrared spectroscopy is in its infancy relative to fMRI and EEG, which have an already substantial literature in the study of PDOC. However, fNIRS is attracting interest in PDOC research as it can provide moderate spatial and temporal resolution of brain data *via* a portable and non-invasive device (please refer to Table 3 for an overall summary of the literature using fNIRS in patients with PDOC or LIS). Therefore, it has potential to improve the accuracy of diagnoses and even provide access to communication devices for more patients than could be achieved with, for example, fMRI alone.

**Table 3:** Summary of the current literature using functional near-infrared spectroscopy (fNIRS) in patients with prolonged disorder of consciousness (PDOC) or locked-in-syndrome (LIS).

Diagnosis	Number of patients	Overview of main results	Reference
PDOC	2—MCS	▪ Functional activation (i.e., [HbO] and [HbR]) during passive and somatosensory stimulation ▪ Weak brain activations during active hand opening and closing	Molteni et al., 2013 (28)

PDOC	5—UWS/VS 11—MCS	▪ Hemispheric differences during motor imagery of squeezing a ball with the right hand ▪ Patients in a minimally conscious state shared fNIRS profiles similar to healthy participants	Kempny et al., 2016 (27)

PDOC	7—UWS/VS 2—MCS	▪ In eight of the nine patients, spinal cord stimulation for 30 s induced sustained cerebral blood volume changes in the prefrontal cortex (an area important in the consciousness system; measured through an increase in [HbT]) ▪ An inter-stimulus interval of 2 min significantly improved amplitudes of the HbT across blocks	Zhang et al., 2018 (29)

LIS	40	▪ The intentions of 23 patients were successfully detected (80% correctly identified) by assigning different mental tasks to “yes” and “no” responses	Naito et al., 2007 (150)

LIS	1	▪ The responses to open sentences were detected by instructing the patient to think “yes” and “no” to several questions ▪ 72% of responses were correctly identified at the bedside	Gallegos-Ayala et al., 2014 (162)

LIS	4	▪ Communication using open sentences was established by instructing the patient to think “yes” and “no” to several questions ▪ For three out of the four patients, classification accuracies exceeded 75%	Chaudhary et al., 2017 (163)

LIS	1	▪ Without any prior training, tennis-playing motor imagery was used successfully by a patient as a proxy to communicate responses to three questions ▪ Results were confirmed by the patient’s residual eye-movement communication channel ▪ Responses were similar to that of healthy participants performing the same task	Abdalmalak et al., 2017 (87)

*MCS, minimally conscious state; UWS, unresponsive wakefulness syndrome; VS, vegetative state*.

Following in the footsteps of fMRI and EEG research in PDOC, the majority of fNIRS research has focused on detecting covert command-following *via* sensorimotor activity during imagined actions. However, for clinical applications, fNIRS is so far insufficiently sensitive to detect task-relevant activation in single-subject data. Furthermore, no fNIRS study has yet differentiated between vegetative and minimally conscious states, suggesting limited diagnostic utility so far. However, as the sensorimotor cortex is easily probed by scalp-based sensors, and often a target for other hemodynamic markers of covert command-following (i.e., fMRI), the PDOC field should commit to developing sensitive fNIRS markers of motor imagery.

Due to the relative infancy of fNIRS, there remains significant work to do in terms of hardware, signal processing, and analyses, especially for those researchers and clinicians who are not experts in optical imaging. For example, the vast majority of fNIRS work in motor imagery has been conducted with less sensitive but simpler continuous wave devices. Further research into the operations of more advanced fNIRS systems (e.g., high channel density frequency domain and time domain devices) and subsequent knowledge transfer to clinical and biomedical science users will enable greater resolution of clinically meaningful brain responses. Indeed, across the broad physical and computational sciences of optical imaging, there has been significant work in improving the sensitivity of the brain tissue sampled, the depth of the measure, and the tools and models used to examine light propagation and detection. Recent successes in combining fNIRS and EEG analyses for BCIs also indicate that the technology is reaching the standards required for clinical applicability in PDOC.

It is clear that for fNIRS to realize its potential in PDOC assessment, research teams must incorporate multidisciplinary expertise in cognition, clinical practice, physical sciences, and computational sciences. With principled paradigms for diagnosing covert awareness in combination with state-of-the-art devices and algorithms for data modeling, and feature extraction/classification, fNIRS, and perhaps more so EEG-fNIRS, has great potential to improve diagnostic accuracy in PDOC and enable patients to communicate their true mental state to the outside world.

## Author Contributions

MR wrote the initial draft of the paper. MR and DC revised the draft. All the authors contributed to subsequent drafts.

## Conflict of Interest Statement

The authors declare that the research was conducted in the absence of any commercial or financial relationships that could be construed as a potential conflict of interest.

## Footnotes

^1^https://www.headway.org.uk/about-brain-injury/further-information/statistics/.
